# A Bibliometric Analysis of Knowledge-Hiding Research

**DOI:** 10.3390/bs12050122

**Published:** 2022-04-21

**Authors:** Qing Xia, Shumin Yan, Heng Li, Kaifeng Duan, Yuliang Zhang

**Affiliations:** 1 School of Economics and Management, Tongji University, Shanghai 200092, China; veraxia2017@tongji.edu.cn (Q.X.); yanshumin@tongji.edu.cn (S.Y.); 2 Department of Building and Real Estate, Hong Kong Polytechnic University, Hung Hom, Kowloon, Hong Kong 999077, China; heng.li@polyu.edu.hk; 3 School of Economics and Management, Fuzhou University, Fuzhou 350108, China; kefee920729@tongji.edu.cn

**Keywords:** knowledge hiding, bibliometric research, publication performance, thematic evolution

## Abstract

Knowledge hiding, defined as an intentional attempt to conceal requested knowledge, has become a hot topic in management and psychology in the last decade. Emerging research has suggested that knowledge hiding is not simply the opposite of knowledge sharing, such that it is crucial to clarify the concept, explore the research progress and development trend of knowledge hiding. Based on 243 relevant articles, a bibliometric analysis of knowledge-hiding research is presented via descriptive, keyword and citation analysis. Results reveal that knowledge-hiding research, mainly focusing on the disciplines of management, business and psychology, is currently in a period of rapid growth, especially in the past two or three years. The systematic review of knowledge-hiding research enables us intuitively to obtain a panoramic view, including publication performance, thematic evolution and most influential topics of the field via a set of science maps, enabling future authors to investigate knowledge hiding and focus their research more effectively.

## 1. Introduction

Effective knowledge management and organizational learning are critical for organizational strategic adaptive abilities and competitive advantage [1,2], and are highly dependent on organizational employees’ knowledge sharing. Even though efforts have been made to enhance knowledge sharing within organizations, employees are still reluctant to share knowledge with other members [3,4]. Empirical evidence has demonstrated that knowledge hiding has serious implications, such as hurting relationships, eliciting negative emotions and threatening psychological safety [5,6,7]. Although knowledge hiding ubiquitously exists among organizational members, rigorous concepts, theory development and empirical research on knowledge hiding have been sporadic and stagnant until recent years, when a formal constructive concept of knowledge hiding was developed [3]. Since then, knowledge hiding has become a stand-alone research topic and scholars have been attracted to the field, contributing to the rapid development of the field in recent years.

Furthermore, some attempts have been made to review knowledge-hiding literature with different goals and focuses. Xiao and Cooke [8] have analyzed 52 articles (33 English articles and 19 Chinese articles) published during 1997 and 2017 to clarify the concept and measures, three widely employed theories and the research findings on knowledge hiding. Connelly, Černe, Dysvik and Skerlavaj [4] have described the five articles that comprised the Journal of Organizational Behavior special issue on knowledge hiding and introduced the overview of the latest developments in knowledge hiding. While these reviews on knowledge hiding contribute to our better understanding of its concepts, theories, research findings and future trends, the existing review papers are qualitative reviews that can be subjective and difficult to replicate.

Bibliometric analysis, a computerized technique to perform metrological and content analyses of the bibliometric data [9], can help overcome some limitations. Relevant tools can automatically identify and extract the information needed and present it in an Excel spreadsheet or a map, and the results are fast, straightforward, consistent and rich [10]. Thus, the present paper attempts to combine the merits of qualitative reviews with computer technology to systematically review the existing knowledge-hiding articles during 1997 and 2020. To be specific, we combined the bibliometrix R-package with VOSviewer software to evaluate the publication performance and identify the intellectual structure of knowledge-hiding research. Crucially, we learnt from the categorizations from previous reviews and integrated the previous categorizations with outputs performed by software tools in our thematic scheme.

Besides making the analyzing process more objective and transparent, we aim to make several additional contributions as follows. First, in our article, we conduct descriptive analysis to reveal the popularity of knowledge-hiding research across time and evaluate the publication performance according to a series of indexes (e.g., h-index, the number of publications, citations, the year of the first publication). In doing so, more detailed information in knowledge-hiding research can be uncovered. Second, we explore the intellectual structure of knowledge-hiding research by content analysis. We perform co-word analysis to generate the initial thematic scheme of the knowledge-hiding research, and then conduct co-citation and historical analyses to examine and complement the initial thematic scheme. With these three key analyses, we make efforts to summarize the research findings on knowledge hiding, thus enabling future authors to investigate knowledge hiding more effectively. Third, compared to past reviews of knowledge hiding, we have a longer study span, and a larger number of and more up-to-date data (243 publications from 1997–31 March 2022). We start from 1997 because it is the earliest available date in the knowledge-hiding research. The longer study span and the up-to-date data contribute to a better understanding of the overview and future directions on knowledge-hiding research.

## 2. Literature Review

Knowledge hiding refers to intentional attempts to withhold or conceal knowledge from another individual [3]. Connelly et al. [3] has identified three types of knowledge hiding: evasive hiding, playing dumb and rationalized hiding. Evasive hiding, which involves deception, means that the hider provides incorrect information or a misleading promise of a complete answer in the future, even though there is no intention to actually provide it or an intention merely to delay as much as possible. Playing dumb also involves some deception and a lack of intention to help, and refers to a situation in which the knowledge hider pretends that he/she does not understand what the requester is talking about and thus achieves the purpose of hiding knowledge. Rationalized hiding does not necessarily involve deception, and refers to a situation in which the knowledge hider is “offering a justification for failing to provide requested knowledge by either suggesting he or she is unable to provide the knowledge requested or blaming another party” ([6] p. 480). Besides evasive hiding, playing dumb and rationalized hiding, Yuan et al. [11] identify bullying hiding as another dimension of knowledge hiding and conceptualize bullying hiding as the negative interference behavior of the requestees based on power and status.

At the same time as Connelly et al. [3] proposed the concept of knowledge hiding, they made it clear that knowledge hiding is distinct from sets of behaviors such as knowledge sharing and counterproductive workplace behavior (CWB). Knowledge hiding is not simply the lack of knowledge sharing. To be specific, a lack of knowledge sharing may be only because of ignorance; however, knowledge hiding is an intentional attempt to conceal knowledge and may be driven by different reasons, such as instrumental factors or laziness. Kang [12] identifies that knowledge sharing and knowledge withholding—being classified into intentional hiding and the unintentional hoarding of knowledge—are separate concepts, according to Herzberg’s two-factor theory. Knowledge hiding is also distinct from CWB. CWB comprises those behaviors “intended to have a detrimental effect on organizations and their members” ([13] p.292), while knowledge hiding is not necessarily destructive such that knowledge hiding (rationalized hiding) may be driven by prosocial motivations, such as preserving confidentiality and protecting the other party’s feelings [3].

## 3. Method

Bibliometric analysis was used in this paper to explore knowledge-hiding research. A bibliometric analysis applies quantitative statistical analysis to publications and provides an objective, quantitative, systematic, transparent and reproducible process [14,15,16]. Descriptive analysis and content analysis are two major bibliometric techniques [17]. Descriptive analysis comprises a series of indexes of publications and journals that help to evaluate the publication performance of individuals and sources. Content analysis, on the other hand, reveals the intellectual structures of the specific subjects, commonly including keywords and citation analyses that detect hot topics, thematic evolution and research focuses. In this work, we used an open-source R-package bibliometrix [14] and VOSviewer [18] to assist in performing a comprehensive bibliometric analysis of knowledge-hiding research.

We first, according to previous bibliometric studies [9,19,20], collected data from the Web of Science Core Collection’s Social Science Citation Index (SSCI) by the Thomson Reuters online database. The SSCI includes 3574 journals that demonstrate high levels of editorial rigor and best practice, according to the Journal Citation Reports (JCR) of 23 March 2022 (https://mjl.clarivate.com/). It has been suggested that the Web of Science has a significant advantage over other databases because it includes social science literature [17,21].

According to previous literature reviews [8,22,23], we searched the titles, abstracts, author keywords and keywords of the publications. The search formula used, according to Xiao and Cooke [8], was: “knowledge hid*” or “knowledge withhold*” or “knowledge hoard*” or “information hid*” or “information withhold*” or “data withhold*” or “partial knowledge sharing”or “knowledge sharing hostile” or “knowledge-sharing hostile” and (publishing date was set from 1 January 1975 to 31 March 2022). Here, “*” means a fuzzy search; the earliest publishing date of SSCI is 1 January 1975, and the search was conducted in 1 April 2022. This search resulted in a preliminary list of 374 publications.

Only English language articles were included, resulting in 370 publications. After that, we restricted results to journal articles, and excluded conference papers, editorials, review papers and revision, yielding 350 articles. Finally, we read and assessed to find the papers focusing on knowledge hiding, and excluded the papers that focused on sharing but merely mention knowledge hiding and those that focused on knowledge hiding in databases such those discussing the hiding of sensitive data and the hiding of sensitive knowledge contained in data. A collection of 243 scientific articles between the earliest available date (1997) and 31 March 2022 were found with these inclusion and exclusion criteria. These 243 records were used as the dataset and were fixed as the basis for bibliometric analysis in this paper.

## 4. Results

### 4.1. Descriptive Analysis

#### 4.1.1. Main Information Regarding the Collection

Table 1 shows the main information of the analyzed collection, which includes the main information about data, keywords, countries, institutions and authorship. The authorship provides rich and valuable information regarding the characteristics of the authors and authors’ collaboration [24,25]. As shown in Table 1, the 243 articles constituting the sample are by 640 authors affiliated with 385 institutions in 47 countries or regions and published in 85 journals.

#### 4.1.2. Annual Number Distribution and Citations

**Figure 1:**
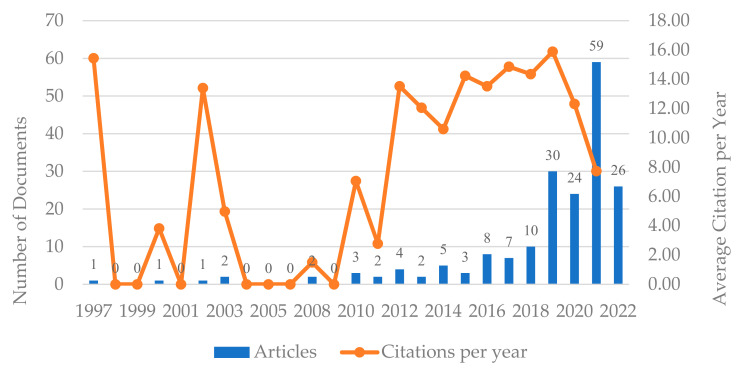
Annual number distribution and citations.

Figure 1 shows the annual number distribution and citations of the 243 articles included in the sample. According to the histogram in Figure 1, the growing pattern between 1997 and 2022 and the chronological distribution show three stages in the knowledge-hiding publication trend. The early days comprise the period from 1997 to 2009. In subsequent years, 2010–2015, publications were scarce. The number of publications increases considerably from 2016 onwards and the trend is upward. The annual growth rate of knowledge-hiding research from 1997 to 2022 is 21.12%, which indicates that the topic of knowledge hiding is increasing in popularity. As for the average citations per year of each article, publications in 2019 have the most average citations,15.889, followed by publications in 1997 [26] (with a citation number of 16.44) and publications in 2017 (with 14.857 average citations).

#### 4.1.3. Most Relevant and Influential Journals

This study identifies 243 articles published in 85 peer-reviewed journals. The Hirsch index (h-index) of each journal is used as the measure to identify the most influential journals in knowledge-hiding research. The H-index, a widely accepted indicator for measuring the research achievement of an author or a journal, is defined as the number of papers of an individual or a journal that have been cited in other papers at least h times [27,28]. Table 2 shows the top 20 ranking journals in terms of h-index. Moreover, the total citations (TC), number of publications (NP) and year of first publication (PY-start) are also revealed. These 20 journals can be viewed as the most relevant and influential sources in knowledge-hiding research. As shown in Table 2, Journal of Knowledge Management has the highest h-index of 21, with 1571 citations, 47 publications and its first publication in 2010; Journal of Organizational Behavior has the second-highest h-index of 8, with 955 citations, 9 publications and its first publication in 2012; Journal of Business Research (with 242 citations, 22 publications and its first publication in 2019) and Management Decision (with 215 citations, 7 publications and its first publication in 2017) have the third-highest h-index of 6.

#### 4.1.4. Leading Authors

**Figure 2:**
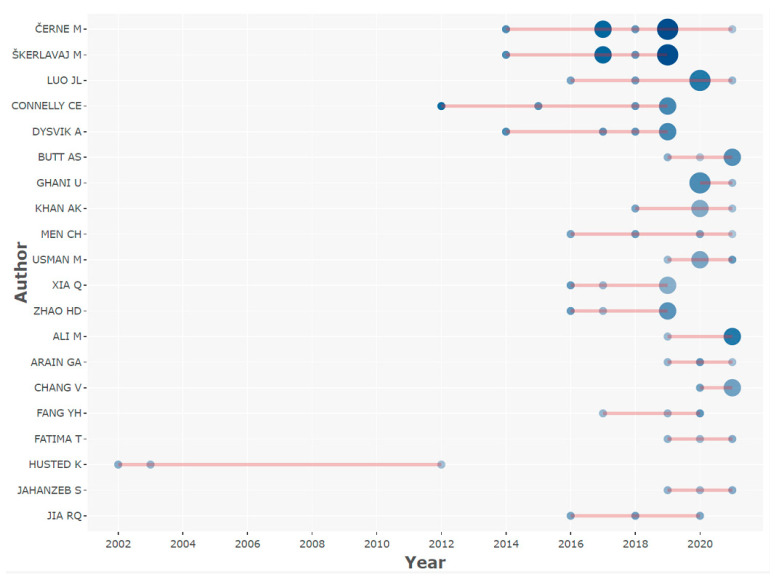
Top 20 authors’ productions over times in knowledge-hiding research field.

The h-index, TC, NP and PY-start are presented in Table 3 to reveal the top 20 influential authors in knowledge-hiding research in terms of h-index. Figure 2 shows their productions over time. In Figure 2, the volume of the spheres is proportional to the NP in each year, while the color depth of the sphere is proportional to TC per year [9]. As shown in Table 3, the top three ranking authors in terms of h-index are Černe M (with 10 publications, an h-index of 7827 citations and their first publication in knowledge-hiding research in 2014), Škerlavaj M (with 7 publications, an h-index of 7817 citations and their first publication in knowledge research in 2014), and Luo JL (with 7 publications, an h-index of 6333 citations and their first publication in knowledge-hiding research in 2016).

### 4.2. Content Analysis

Keyword and citation analyses were applied to identify the research contents of knowledge hiding. In this section, Bibliometrix and VOSview are applied in combination to visualize the network maps concerning keyword co-occurrence and citation analyses [14,29,30,31].

#### 4.2.1. Co-Word Analysis

**Figure 3:**
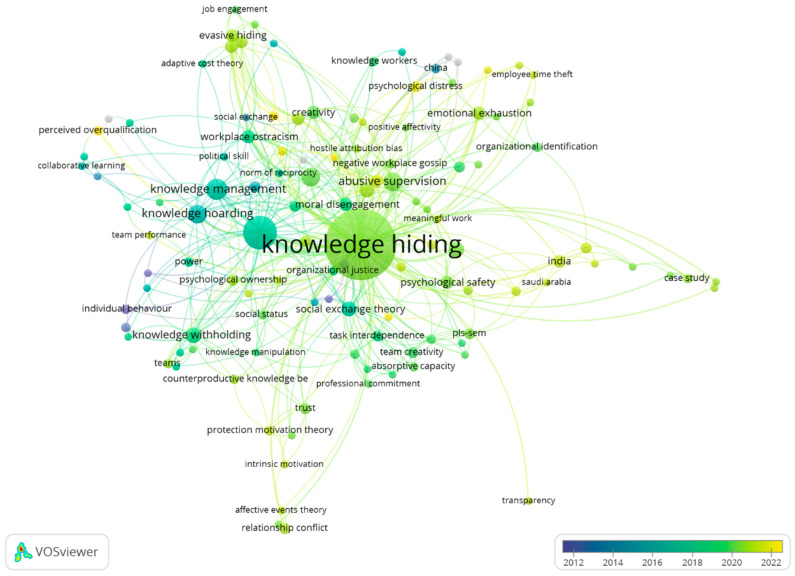
Keyword co-occurrence network.

Keywords are typically used by authors to describe the research content generally; thus, identifying the thematic scheme of a specific subject based on co-occurrence is plausible [14,32,33]. We applied VOSviewer to output keywords to a co-occurrence network of the collection with time information (see Figure 3). The authors’ keywords were used to retain the authors’ meaning. The distance between two keywords in the co-occurrence network reflects their link strength and relatedness, such that the shorter the distance between the two, the stronger their relatedness [34]. Moreover, the color of each node (keyword) in the co-occurrence network reveals the average publication year, the mean of the publication years of all the documents with keywords in their titles or abstracts. Keywords that appear more towards 2012 are shown in dark blue, and those that appear more towards 2022 are shown in yellow. Furthermore, the average publication year of knowledge hiding in the collection is 2019, which reveals that knowledge hiding is an emerging research topic and has a growing demand that needs to be further explored.

Based on the keyword co-occurrence network, the existing review literature [22,35], and our reading of each article in the network (an example of the summarizing process is shown in Table 4), five major topics were initially identified for the research interests related to knowledge hiding in this paper. They are: (1) concept development, (2) theoretical underpinning, (3) methods/analyzing technology, (4) antecedents, (5) outcomes and (6) context factors. Table 5 shows the major research interests in knowledge hiding.

Table 5 reveals that keywords related to the “concept development” of knowledge hiding are knowledge management, knowledge sharing, knowledge withholding, knowledge hoarding, counterproductive knowledge work behavior and workplace bullying. The literature on knowledge hiding in the collection has been developed from knowledge management. Early studies focused on data withholding in academia [26,45] Subsequently, interest in knowledge-sharing hostility [46,47], knowledge withholding [48,49] and knowledge hoarding [49,50] has been increasing. Connelly, Zweig, Webster and Trougakos [3] formally constructed the concept of knowledge hiding. Since then, knowledge hiding has become a stand-alone research topic and has developed rapidly.

The theoretical underpinnings of knowledge hiding mainly include social exchange theory [51,52], social cognitive theory [48,53], psychological ownership theory [36,37], conservation of resource theory [54,55,56], self-determination theory [57,58,59], affective events theory [60,61,62] and self-determination theory [57,63]. The methods/analysis technology used in knowledge-hiding research mainly include case studies [64,65,66], pls-SEM [67,68], experiment analyses [5,51,69], multilevel analyses [37,69,70,71,72], ground theory approaches [73], fuzzy-set qualitative comparative analyses [74] and diary studies [61,75].

The antecedents of knowledge hiding can be divided into five aspects: knowledge, job, individual, interpersonal/team and organizational characteristics. Specifically, the knowledge characteristics mainly include knowledge complexity, work-relatedness and implicitness [3,11]. The job characteristics mainly include task interdependence, job autonomy, time pressure and task conflict [44,57,76]. The influencing factors on an individual level are focused on personality traits, such as the dark triad and negative affectivity [43,77]; abilities, such as knowledge-sharing self-efficacy, overqualification and workplace status [78,79,80]; motivation, such as knowledge-sharing motivation (e.g., autonomous motivation and external motivation)and goal orientations [57,81]; attitude, such as psychological ownership and organizational identity [36,82]; psychological states, such as psychological safety and psychological entitlement [72,83] and emotions, such as envy and anger [61,78,80]. The interpersonal/team characteristics mainly include interpersonal relationships, such as the leader–member exchange, interpersonal distrust and interpersonal conflict [3,82,84,85]; leadership or leader behavior, such as ethical leadership and abusive supervision [86,87]; interpersonal mistreatment, such as workplace ostracism and negative gossip [39,54,88]; and interpersonal behavior, such as leader-signaled knowledge hiding and coworkers’ past opportunistic behaviors [89,90]. The organizational characteristics mainly include the climate, such as the knowledge-sharing climate; organizational justice; and communication visibility [3,91,92].

The outcomes of knowledge hiding mainly focus on creativity, performance, interpersonal relationships, innovative work behavior and organizational citizenship behavior (OCB). It has been linked to reduced levels of individual and team creativity [51,52,71,93], team performance [1,94] and innovative work behavior or innovation [70,95]; it also hurts interpersonal relationships [6] and results in greater interpersonal distrust [68]. Finally, context factors mainly refer to the moderators demonstrated in knowledge-hiding empirical studies. The context factors mainly include the motivational and forgiveness climates, decision autonomy, cross-functionality, task interdependence, moral disengagement, local and foreign workers and gender difference according to the keyword co-occurrence network [39,43,51,54,70].

#### 4.2.2. Co-Citation Analysis

**Figure 4:**
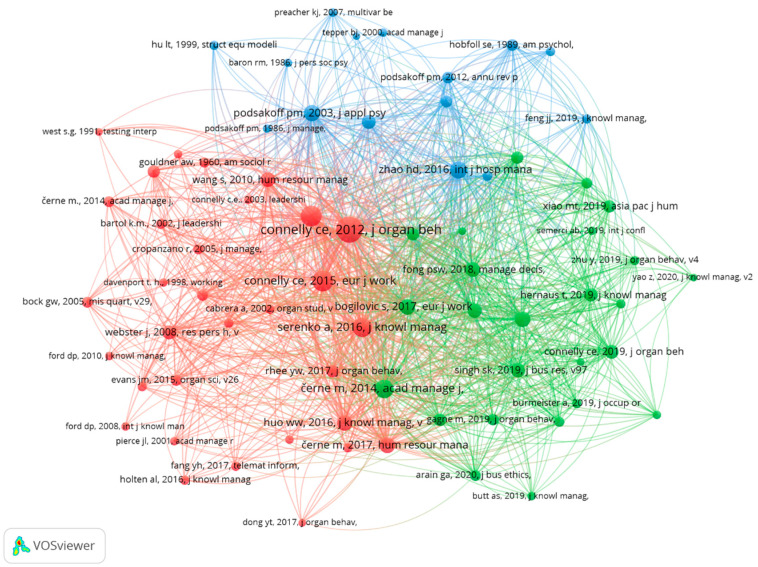
Co-citation analysis of highly cited references.

A total of 11,173 references were cited by the collected 243 papers in knowledge-hiding research. The co-citation of two publications occurs when both are cited in a third publication, and the more the two are cited, the more similarities between them can be assumed [34]. VOSviewer was applied to analyze and visualize the co-citations of the cited references in the knowledge-hiding research. The minimum number of citations of a cited reference was set as 20 (the default value from VOSviewer), and of the 11,173 cited references, 73 met the threshold. The results of the co-citation analysis are shown in Figure 4. Each circle represents a publication; the larger size the circle is, the more the publication has been cited in the collection; circles sharing the same color illustrate a similar topic shared by these publications; the distance between two circles reveals the strength of the relationship and the similarity between two publications. Moreover, the co-citation network shows how the references cited in the collection cluster together.

As shown in Figure 4, three clusters are clearly distinguished from each other, in which each cluster indicates a subfield of the knowledge-hiding research: a red (left), a green (right) and a blue (upper). The three clusters are separated from each other. On the base of the examination of the titles and abstracts of all publications and the full texts of the top 10 cited references (see Table 6) in the three clusters, an appropriate label could be assigned to each of them.

The red cluster represents the subfield of transition from knowledge sharing to knowledge hiding and knowledge-hiding research findings. Not only is knowledge sharing [2] included in this cluster, but also, amongst others, counterproductive knowledge behavior [38], the first time “knowledge hiding” is used as a multidimensional construct to capture the dyadic situations where work-related knowledge is requested by one employee to another [3], and antecedents such as interpersonal distrust and psychological ownership [3,36,37]. The green cluster mainly focuses on findings related to knowledge hiding in the most recent five years, especifially in 2019, including antecedents, such as time presure; performance-prove goal orientation and leader–member exchange [44,81,82]; outcomes, such as thriving; self-conscious moral emotions (shame and guilt); organizational citizenship behavior; and team performance [5,7,94]. The blue cluster mainly focuses on one of the common method biases and time-lagged research design as applied to knowledge hiding [39,96].

The highly cited references used in publications on knowledge hiding (see Table 6) can be divided into two groups: (1) a group of references that are a part of the 243 publications in the collection, and (2) a group of references spanning other research domains that conceptually overlap with and are potentially relevant to knowledge hiding. By examining the second group of references, the important influences of other related topics on knowledge-hiding research can be identified. “Knowledge sharing: A review and directions for future research” from Wang and Noe [2] does not belong to the main field of knowledge hiding, but involves a highly related concept that provides a theoretical basis and comparative study for knowledge-hiding research. “Common method biases in behavioral research: a critical review of the literature and recommended remedies” from Podsakoff et al. [96] also does not belong to the main domain of knowledge-hiding research, but bears significance in influencing knowledge-hiding research by discussing measurement.

#### 4.2.3. Historical Analysis

**Figure 5:**
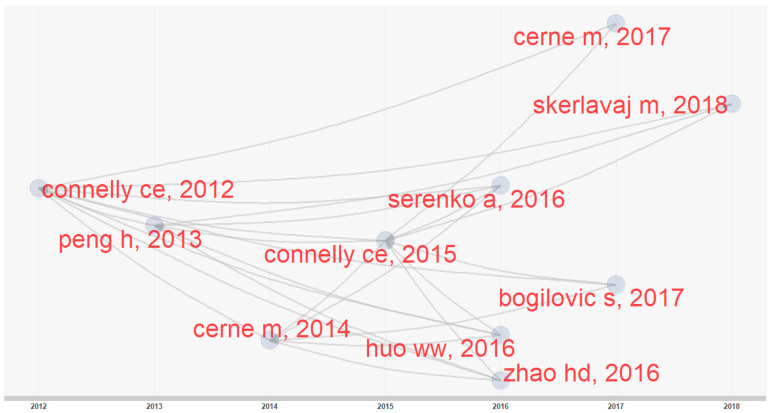
Historical mapping of the top 10 locally cited documents.

Our historical analysis is a chronological map of the most relevant and highly locally cited publications in the bibliographic collection [14,97]. In the historical map, produced by bibliometrix, each node represents a publication included in the analyzed collection, each edge represents a direct citation between two publications and the horizontal axis represents publication years. The historiograph network (see Figure 5) for the top 10 locally cited documents in the knowledge-hiding collection reveals one research path with 10 nodes.

Digging into the full texts of these 10 key documents can help comprehend the evolution of the hot topic of knowledge-hiding. The earliest seed of this research path is the publication from Connelly, Zweig, Webster and Trougakos [3], which puts forward the concept of knowledge hiding for the first time, thus laying a theoretical foundation for the subsequent research on knowledge hiding. All the other articles in the top 10 local citations have been cited in this paper (see Figure 5) and mainly investigated the potential antecedents [36,37,38,39,44] and outcomes [6,38,51,70,93] of knowledge hiding. Moreover, much research has examined knowledge hiding as an overall construct, and only a few of them have explored the three-dimensional structure of knowledge hiding [6,37,39].

**Figure 6:**
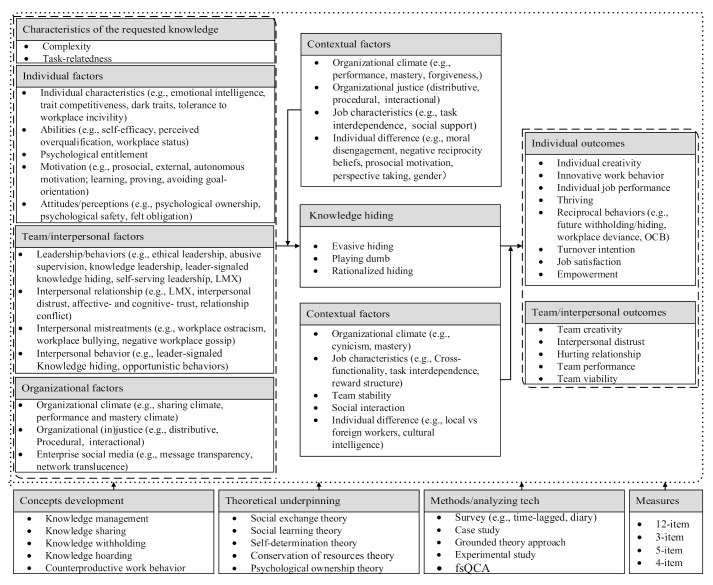
A framework of knowledge-hiding research according to content analysis.

Taken together, based on the outcome of co-word, co-citation and historical analyses and the frameworks from Connelly, Zweig, Webster and Trougakos [3] as well as Xiao and Cooke [8], seven topics (see Figure 6) were identified for knowledge-hiding research in this paper. They are: (1) concept development, (2) theoretical underpinning, (3) methods/analyzing technology, (4) measurements, (5) antecedents, (6) outcomes and (7) context factors.

Source: extended and developed from Connelly et al. (2012) [3] and Xiao and Cooke (2019) [8].

## 5. Future Research Directions

As discussed above, the extant body of literature on knowledge-hiding research has contributed to advance our understanding of knowledge hiding in organizations. Nonetheless, additional research to extend the literature of knowledge hiding is needed. In this section, thus, we identify several interrelated research directions based on the framework from Xiao and Cooke [8] and the prior literature review on knowledge hiding (see Table 7).

### 5.1. Theoretical Opportunities

Although there exist several measures (one multidimensional scale and many other unidimensional scales) of knowledge hiding at individual level, almost the unidimensional scales “might lack the capability to reflect unique characteristics of knowledge hiding such as intentionally” [8]. Furthermore, the only multidimensional scale, from [3], was developed based on the Western workplace context. Thus, further verification of the measures of knowledge hiding is needed to reflect the unique characteristics of knowledge hiding and to examine possible cultural differences in knowledge-hiding measures. Apart from knowledge hiding at an individual/dyadic level, only few studies have investigated team knowledge hiding [69]. Moreover, the measure of team knowledge hiding was self-reported and adapted from the multidimensional 12-item scale developed by [3], which failed to distinguish team knowledge hiding from individual knowledge hiding. Future research may benefit from identifying the theoretical and methodological validity of team knowledge hiding and developing corresponding team knowledge-hiding scales.

While the existing literature has advanced our understanding of how knowledge hiding develops and impacts outcomes from perspectives of social exchange, social cognition, psychological ownership, conservation of resources and self-determination theories [3,36,51,56,57,68,98], additional efforts may be needed from other theoretical perspectives such as affective events theory and social network theory to improve the understanding of the emotional process of and the specific dyadic nature of knowledge hiding, respectively. Furthermore, much of the existing research has described knowledge hiding as a unitary construct; only few have explored the three-dimensional structure of knowledge hiding [5,6,37,39]. According to the statement from Connelly, Černe, Dysvik and Skerlavaj [4], “it is best understood as consisting of three different facets” ([4] p. 780). Thus, future research may examine one or each facet of knowledge hiding separately if the underpinning theory suggests that only one facet of knowledge hiding is of interest or all three of them if there may be an interesting interplay between the different dimensions.

### 5.2. Methodological Opportunities

Extant research has predominantly investigated knowledge hiding at the individual level; few studies have employed a multilevel approach [4,8]. Although several studies have shown that knowledge hiding may hurt team creativity and performance [71,93,94,99] and one study has investigated the influence of leader–member exchange on knowledge hiding in teams from a social exchange theory perspective [69], the construct of knowledge hiding in teams has not been well documented. Specifically, it remains unclear that why team members actively withhold or conceal knowledge from each other in the face of a specific knowledge request and how knowledge hiding in teams may impact team members as well as team and organizational work-related outcomes.

Except for single-level analysis (either individual or team level), cross-level analysis may be a way to help better understand the cross-level interactions influencing knowledge hiding. Furthermore, only two studies to date have taken a within-person approach to investigate knowledge hiding [61,75]; more attention should be given to an experience-sampling approach, to capture the episodic/event-related nature and the dynamic interactive process of knowledge hiding and to examine the within-person variation in knowledge hiding; to a social network approach, to investigate the dyadic nature of knowledge hiding; to a latent profile approach, to identify naturally occurring profiles of knowledge hiding; and to a configurational approach, to investigate how different combinations of factors lead to knowledge hiding.

## 6. Discussion

Knowledge sharing has been taken as one of the most key elements of organizations’ achieving sustainability and competitive advantages [1,20]. However, employees are still reluctant to share knowledge with other members, and may even deliberately hide or hoard knowledge through various strategies involving euphemism and obscurity [5]. Even if other people in the organization request such knowledge, employees may intentionally conceal or withhold knowledge from the requestor [3]. Although knowledge hiding exists in almost all organizations, it had not been paid enough attention by theorists until recently, when it gained the attention of scholars and developed into a frontier topic of organizational behavior research [3,22]. Based on publications from 1997 to 31 March 2022, we conducted a bibliometric analysis of knowledge hiding to capture more comprehensive information on this stand-alone research topic in knowledge management [22].

In line with previous literature reviews on knowledge hiding [20,100], our keyword co-occurrence and co-citation analyses demonstrate that the concept of knowledge hiding has mostly been developed from knowledge sharing, knowledge-management behaviors, counterproductive work behavior and social exchange [2,3,49,101]. Existing literature has conceptually and empirically identified and assessed the potential similarities and differences between knowledge hiding and knowledge sharing and knowledge hoarding. Webster et al. [49] have demonstrated that knowledge hiding and hoarding represent two different types of knowledge withholding, where knowledge hiding means the concealment of the requested knowledge and knowledge hoarding means the accumulation of knowledge. Kang [12], based on two-factor theory, holds that knowledge withholding includes intentional knowledge hiding and unintentional knowledge hoarding.

As for the similarities and differences between knowledge hiding and sharing, more and more empirical research has included knowledge sharing and hiding within the same study, and has provided their discriminant validity. For example, Rhee and Choi [52] empirically examine the influence of dispositional goal orientations on knowledge management behaviors (knowledge sharing, hiding and manipulation), such that learning and avoiding goal orientation increase both knowledge sharing and manipulation, while proving goal orientation increase knowledge hiding and manipulation. However, little empirical research has investigated both knowledge hiding and hoarding [102], demanding further research in the future to provide evidence-based clarification of knowledge hiding and hoarding and their discriminant validity.

According to content analysis (co-word, co-citation and historical analyses) of knowledge hiding and our interrelated research directions based on the framework from Xiao and Cooke [8], many publications on knowledge hiding, in the last five years, are inspired by the future outlook component of existing research. For an example, Xia et al. [61] and Venz et al. [75] have collected longitudinal data and taken a within-person approach to capture the dynamic process of knowledge hiding, responding to calls from Connelly et al. [4]. Li et al. [80] have investigated the impact of perceived overqualification on knowledge hiding on a dyadic level, Butt and colleges [64,66] have undertaken multiple case studies to qualitatively identify strategies to mitigate knowledge hiding, and Good et al. [60] have investigated the influence of organizational social activities on knowledge management behaviors from affective events perspective to respond to calls from Connelly et al. [4] and Xiao and Cooke [8]. Consequently, future research could further knowledge-hiding research based on or combined with existing the overviews of knowledge-hiding research.

## 7. Limitations and Conclusions

The present study has some limitations. Firstly, only journal articles from the Web of Science Core Collection’s Social Science Citation Index database are included in the analysis collection. Even though Web of Science is one of the largest global databases with high levels of editorial rigor and best practice publications, it does not include all publications on knowledge hiding. Future research may benefit from combined databases, including EBSCO, Scopus, JSTOR, etc. The second limitation involves the retrieval code for collection. We applied keywords from Xiao and Cooke [8] to retrieve articles related to knowledge hiding, which provided a certain theoretical basis for our retrieval strategy. However, these keywords contained some other concepts related to knowledge hiding (e.g., knowledge hoarding, hostile knowledge sharing), which may lead to the generalization of concepts. It should be mentioned that we conducted a manual check on the title, abstract and keyword fields of retrieved journal articles before analyzing them to exclude irrelevant articles, which may to some extent help make up for this limitation.

To conclude, despite the rapid development during the last five years, it can still be clearly found that knowledge hiding is a rather young research topic and needs further investigation [20,100,103]. Building upon the overview of knowledge hiding from Xiao and Cooke [8], and using a combination of the bibliometrix R-package with VOSviewer software to evaluate publication performance and identify the intellectual structure of knowledge-hiding research, our descriptive analysis of the updated samples shows that knowledge hiding mainly focuses on the annual number distribution and citations, most relevant and influential journals, and authors to evaluate the publication performance. The annual number distribution of publications indicates a growing pattern with an annual growth rate of 21.12% from 1997 to 2022. The Journal of Knowledge Management is the most relevant and influential journal in knowledge-hiding research. The leading journals mainly focus on knowledge management, organizational behavior and psychology, and future efforts should be directed to more multidisciplinary research. Černe M and Škerlavaj M are the most prominent researchers in knowledge-hiding research, followed by Luo JL, Zhao HD and Connelly CE. As regards the intellectual structure of knowledge-hiding research, keyword co-occurrence, co-citation and historical analyses are combined to identify the major research interests in knowledge hiding. Seven sub-topics of knowledge-hiding research have been identified: concept development, measurements, theoretical underpinning, methods, antecedents, outcomes and context factors.

## Figures and Tables

**Table 1:** Summary of general results.

Description		Results	Description		Results
Main information about data	Journals	85	Authors	Authors	640
	Average years from publication	3.32		Author appearances	829
	Average citations per documents	29.34		Authors of single-authored documents	20
	Average citations per year per documents	7.08		Authors of multi-authored documents	620
	References	11173	Authors collaboration	Single-authored documents	22
Document contents	Keywords plus	642		Documents per author	0.38
	Author’s keywords	807		Authors per document	2.63
Countries/regions		47		Co-authors per documents	3.39
Institutions		385		Collaboration index	2.8

Notes: Documents per author = Documents/Author; Authors per Document = Authors/Document; Co-Authors per documents = Author Appearances/Documents; Collaboration Index = Authors of multi-authored documents/Multi-authored documents [24,25].

**Table 2:** Top 20 influential journals.

Source	h-Index	TC	NP	PY-Start
Journal of Knowledge Management	21	1571	47	2010
Journal of Organizational Behavior	8	955	9	2012
Journal of Business Research	6	242	22	2019
Management Decision	6	215	7	2017
Knowledge Management Research & Practice	5	114	11	2008
Leadership & Organization Development Journal	5	130	6	2014
Computers in Human Behavior	4	97	5	2011
Frontiers in Psychology	3	50	21	2018
Journal of Business Ethics	3	174	5	2019
European Journal of Work and Organizational Psychology	3	351	4	2015
International Journal of Hospitality Management	3	183	4	2016
Organization Science	3	145	4	2010
Sustainability	2	36	5	2019
International Journal of Conflict Management	2	52	4	2019
Asian Business & Management	2	35	3	2021
Current Psychology	2	9	3	2021
International Journal of Contemporary Hospitality Management	2	7	3	2021
Human Relations	2	45	2	2011
Information & Management	2	124	2	2010
Interactive Learning Environments	2	35	2	2020
International Journal of Information Management	2	148	2	2018
Journal of Managerial Psychology	2	39	2	2020
Journal of Nursing Management	2	34	2	2019

Note: TC represents total citations. NP represents the number of publications. PY-start represents the year of the first publication.

**Table 3:** Top 20 influential authors.

Author	Institutions	Countries (Regions)	h-Index	TC	NP	PY-Start
Černe M	University of Ljubljana	Slovenia	7	827	10	2014
Škerlavaj M	BI Norwegian Business School	Norway	7	817	7	2014
Luo JL	Tongji University	China	6	333	7	2016
Zhao HD	Shanghai University	China	5	301	9	2016
Connelly CE	McMaster University	Canada	5	856	5	2012
Dysvik A	BI Norwegian Business School	Norway	5	597	5	2014
Ghani U	Zhejiang University	China	4	96	5	2020
Khan AK	United Arab Emirates University	United Arab Emirates	4	132	5	2018
Xia Q	Tongji University	China	4	237	5	2016
Butt AS	American University of Ras Al Khaimah	United Arab Emirates	4	99	4	2019
UsmanM	COMSATS University Islamabad	Pakistan	4	116	4	2019
Arain GA	American University of Ras Al Khaimah	United Arab Emirates	3	111	4	2019
Fatima T	NFC IET	Pakistan	3	86	4	2019
Jahanzeb S	Memorial University of Newfoundland	Canada	3	86	4	2019
Men CH	Shandong University	China	3	260	4	2016
Ali M	King Abdulaziz University	Saudi Arabia	3	96	3	2019
Fang YH	Tamkang University	Taiwan	3	147	3	2017
Huo WW	Shanghai University	China	3	164	3	2016
Husted K	University of Auckland	New Zealand	3	492	3	2002
Jia RQ	Tongji University	China	3	257	3	2016
Koay KY	Sunway University	Malaysia	3	42	3	2018
Michailova S	Copenhagen Business School	Denmark	3	492	3	2002
Zhai XS	Zhejiang University	China	3	59	3	2020

**Table 4:** An example of the summarizing of empirical knowledge-hiding studies.

Publication	Theoretical Perspective	Method	Antecedents (Significance)
Connelly et al. [3]	Social exchange theory interdependence theory	Study 1: event-based experience sampling study and qualitative interviews Study 2: survey Study 3: survey	EH/PD/RH (+/+/+) Interpersonal distrust (S/S/S) (+/+/+) Knowledge complexity (S/N/N) (+/+/−) Task related knowledge (S/N/S) (−/+/−) Knowledge sharing climate (S/N/N)
Peng [36]	Psychological ownership theory	Time-lagged survey (three times)	(+) Knowledge-based psychological ownership (S) (+) Territoriality (S)
Huo et al. [37]	Psychological ownership theory	Time-lagged survey (two times)	EH/PD/RH (+/+/+) Psychological ownership (S/S/S) (+/+/+) Territoriality (S/S/S)
Serenko and Bontis [38]	Social exchange theory	Cross-sectional survey	Intro-organizational KH (+) KM system (N) (+) Knowledge policies (N) (−) Positive culture (S) (+) Involuntary turnover rate (S) (−) Compensation per full-time equivalent (S)
Zhao et al. [39]	Norms of reciprocity	Time-lagged survey (two times)	EH/PD/RH (+/+/+) Workplace ostracism (S/S/N)
Fang [40]	Coping theory	Cross-sectional survey	(+) Self-referenced fear and (S) (+) Other-referenced fear (S) (−) Guilt (S)
Aljawarneh and Atan [41]	Conservation of resources theory Psychological ownership theory	Time-lagged survey (two times)	(+) Tolerance to workplace incivility (S) (+) Employee cynicism (S)
Khalid et al. [42]	Displaced aggression theory Social exchange theory	Time-lagged survey (three times)	(+) Abusive supervision (S) (−) Interpersonal Justice (S)
Pan et al. [43]	Psychological contract theory	Matched-pair data (coworker-employee)	EH/PD/RH (+/+/+) Machiavellianism (S/S/S) (+/+/+) Narcissism (S/S/S) (+/+/+) Psychopathy (S/S/S)
Škerlavaj et al. [44]	Conservation of resources theory	Study 1: Time-lagged survey (two times) Study 2: Lab experiment	Study 1 (+) Time pressure (S) (−) Pro-social motivation (S) Study 2 (+) Time pressure (S)

Note: KH represents knowledge hiding; EH represents evasive hiding; PD represents playing dumb; RH represents rationalized hiding; (+) represents positive related; (−) represents negative related; (S) represent significant at least *p* < 0.05; (N) represents non-significant at *p* < 0.05.

**Table 5:** Identified research topic.

Topic		Related High-Frequency Keywords
Concept development		Knowledge sharing, knowledge management, knowledge withholding, knowledge hoarding, counterproductive knowledge work behavior, workplace bullying, evasive hiding
Theoretical underpinning		Social exchange theory, social cognitive theory, psychological ownership theory, conservation of resource theory, self-determination theory, affective events theory
Methods/analyzing technology		Case study, pls-SEM, experiment analysis, multilevel analysis, ground theory approach, fuzzy-set qualitative comparative analysis, diary study
Antecedents	Knowledge characteristics	Complexity, work-relatedness, implicitness
	Job characteristics	Job autonomy, task interdependence, time pressure, task conflict, task complexity
	Individual characteristics	Dark triad, psychological ownership, goal orientation, territoriality, anger, motivation, psychological contract breach, professional commitment, emotional exhaustion, psychological safety
	Interpersonal/team characteristics	Workplace ostracism, interpersonal distrust, ethical leadership, abusive supervision, task/relationship conflict, collaborative learning
	Organizational characteristics	Sharing climate, competitive climate, organizational injustice
Outcomes		Creativity, performance, interpersonal relationship, innovative work behavior, OCB, innovation
Context factors		motivational climate, forgiveness climate, decision autonomy, cross-functionality, task interdependence, gender difference, moral disengagement, local and foreign workers, perceived overqualification

**Table 6:** Top 10 highly cited references in co-citation network.

Rank	Reference	Local Citations	Cluster
1	Knowledge hiding in organizations [3]	204	red
2	How perpetrators and targets construe knowledge hiding in organizations [6]	128	red
3	Why and when do people hide knowledge? [36]	125	red
4	Understanding counterproductive knowledge behavior: antecedents and consequences of intra-organizational knowledge hiding [38]	106	red
5	What goes around comes around: knowledge hiding, perceived motivational climate and creativity [51]	103	green
6	Workplace ostracism and knowledge hiding in service organizations [39]	84	blue
7	Common method biases in behavioral research: a critical review of the literature and recommended remedies [96]	81	blue
8	The role of multilevel synergistic interplay among team mastery climate, knowledge hiding and job characteristics in stimulating innovative work behavior [70]	70	red
9	Hiding behind a mask? Cultural intelligence, knowledge hiding and individual and team creativity [93]	68	green
9	Antecedents and intervention mechanisms: a multi-level study of R&D team’s knowledge-hiding behavior [37]	68	red
9	Tell me if you can: time pressure, prosocial motivation, perspective taking and knowledge hiding [44]	68	green
10	Knowledge sharing: a review and directions for future research [2]	62	red

**Table 7:** A summary of directions for future research on knowledge hiding.

Future Opportunities	Aspects	Indicative Future Research Orientations
Theoretical opportunities	Conceptualization	Further verify the measures of knowledge hiding to reflect unique characteristics of knowledge hiding
		Enrich the theoretical and methodological validity of knowledge hiding in teams based on or compared to Babic et al.’ s (2019) research of knowledge hiding in teams
		Examine one facet of or each facet of knowledge hiding separately if the underpinning theory suggests that only one facet of knowledge hiding is of interest or that three may be an interesting interplay between the different dimensions
	Alternative theoretical perspectives	Use communication theory and social network theory to improve the understanding of the specific dyadic nature of knowledge hiding
Methodological opportunities	Levels of analysis	More studies at within-person, dyadic, team and organizational levels
	Data collection	Collect longitudinal or daily data to capture the dynamic process of knowledge hiding
		Collect roster or nominate data to capture dyadic interactions between requestors and requestees
	Alternative methods	Use an experience sampling approach to capture the episodic/event related nature of knowledge hiding and to examine the within-person variation in knowledge hiding
		Use a social network approach to investigate the dyadic nature of knowledge hiding
		Use a latent profile approach to identify naturally occurring profiles of knowledge hiding
		Use a configurational approach to investigate how different combinations of factors leads to knowledge hiding
	Cross-cultural perspectives	More studies adopt a cross-cultural comparative perspective to identify cultural differences in knowledge hiding
	Contexts	Broaden the research context to include social media community, industrial and sociocultural contexts

Source: extended and developed from Xiao and Cooke [8].
